# Managing hepatocellular carcinoma across the stages: efficacy and outcomes of stereotactic body radiotherapy: A retrospective study

**DOI:** 10.1007/s00066-024-02235-5

**Published:** 2024-04-30

**Authors:** Ahmed Allam Mohamed, Marie-Luise Berres, Philipp Bruners, Sven Arke Lang, Christian Trautwein, Georg Wiltberger, Alexandra Barabasch, Michael Eble

**Affiliations:** 1 Radiation Oncology Department, University Hospital RWTH Aachen, Pauwelsstraße 30, 52074 Aachen, Germany; 2 Gastroenterology, Hepatology and infectious Diseases Department, University Hospital RWTH Aachen, Aachen, Germany; 3 Diagnostic and IInterventional Radiology Department, University Hospital RWTH Aachen, Aachen, Germany; 4 Visceral and Transplantation Surgery Department, University Hospital RWTH Aachen, Aachen, Germany; 5 Site: Aachen, Center for Integrated Oncology Aachen, Bonn, Cologne and Duesseldorf (CIO ABCD), Aachen, Germany

**Keywords:** Primary liver cancer, Local ablative therapy, Non-invasive oncologic intervention, High-dose radiation therapy, Precision Oncology

## Abstract

**Purpose:**

Hepatocellular carcinoma (HCC) poses a unique challenge due to its predilection for developing on compromised livers, often limiting surgical options. Stereotactic body radiotherapy (SBRT) has emerged as a promising local treatment modality for HCC. This study aims to assess the effectiveness of SBRT in HCC patients not suitable for surgery, focusing on local control, optimal radiation dosing, and prognostic factors.

**Methods:**

In this retrospective analysis, 52 HCC patients treated with SBRT were examined. The study assessed local control, progression-free survival (PFS), and overall survival (OS) while conducting dosimetric analyses. The relationship between mean liver dose and Child–Pugh score (CPS) progression was also explored.

**Results:**

SBRT demonstrated 93.4% freedom from local progression (FFLP) at 12 months. Notably, a near minimum dose (D98%) below 61 Gy as an equivalent dose in 2‑Gy fractions with α/β 10 Gy (EQD2_α/β10_) was associated with reduced FFLP (*p*-value 0.034). Logistic regression analysis revealed a dose–response relationship for FFLP and D98% with 95% and 98% probability of FFLP at a dose of 56.9 and 73.1 Gy, respectively. The study observed OS rates of 63.7% at 1 year and 34.3% at 3 years. Patients with portal vein tumor thrombus (PVTT) and larger tumors (≥ 37 cm^3^) experienced decreased PFS and OS. Multivariate analysis identified PVTT, larger tumor volume, and performance status as independent predictors of reduced OS. Notably, classical radiation-induced disease (cRILD) was absent, but nonclassical (nc) RILD occurred in 7.7% of patients. Regression analysis linked a mean EQD2_α/β3–8_ dose to the liver (12.8–12.6) with a 10% likelihood of ncRILD.

**Conclusion:**

SBRT offers a compelling option for achieving high local control and promising survival outcomes in HCC. The study supports a radiation dose range of 61–73.1 Gy, coupled with a mean liver dose under 12.6–12.8 Gy as EQD2, to achieve favorable FFLP rates, with acceptable toxicity rates.

**Supplementary Information:**

The online version of this article (10.1007/s00066-024-02235-5) contains supplementary material, which is available to authorized users.

## Introduction

Liver cancer, ranking as the 6th most prevalent malignancy globally and standing as the third leading cause of cancer-related death, is a challenging malignancy and is surpassed only by lung and colorectal cancer [1]. Hepatocellular carcinoma (HCC) is the most common primary liver cancer, accounting for > 80% of cases [2]. Traditionally, surgical resection and orthotopic liver transplantation have been considered the cornerstone of curative interventions for HCC, delivering survival rates exceeding 5 years in cases of early stage HCC [3]. However, their applicability is restricted by stringent criteria. A common predicament is the unsuitability of certain patients for surgical intervention, primarily due to poor hepatic reserve, concurrent comorbidities, or presenting in an advanced stage [4].

In response to these limitations, locoregional therapies such as radiofrequency ablation (RFA), microwave ablation (MWA), or transarterial arterial chemoembolization (TACE) have been employed as minimally invasive alternatives in early and intermediate stages [3].

Meanwhile, the recent advancement in radiation therapy technology has enabled the precise delivery of lethal high radiation doses to localized tumors in one or a few fractions [5]. Consequently, SBRT has gained widespread acceptance as a treatment modality for various primary tumors, including those in the lungs, prostate, liver, and pancreas, or as an ablative tool for various metastatic sites in oligometastatic disease [6–10]. An expanding body of evidence supports the safe and effective application of SBRT in diverse stages in HCC, including early, intermediate, and advanced stages or in the setting of planned orthoptic liver transplantation as a bridging therapy, yielding impressive high control rates of 70–100% [8, 11–16].

Nevertheless, ongoing debates regarding the use of SBRT in HCC warrant further exploration. These include determining the optimal radiation dose that can achieve effective local control while minimizing additional radiation exposure to the cirrhotic liver tissue. In addition, there is a need to identify specific patient subsets that may derive substantial benefit from the high local control rates offered by SBRT and those subsets at higher risk for hepatic and extrahepatic progression who might need or benefit more from combined therapeutic approaches.

In the current retrospective study, we aimed to report on the single institution experience of stereotactic body radiotherapy in HCC, emphasizing local control outcomes as well as the overall and progression-free survivals. Additionally, we aim to determine the optimal radiation dose for HCC that achieves a high control rate while maintaining an acceptably low toxicity profile for SBRT. Finally, we also aimed to investigate the prognostic factors that influence survival to enable us to define suitable candidates for combined therapies in the future.

## Materials and methods

Following approval from the local ethics committee (RWTH Aachen University, Faculty of Medicine, EK 23–264), we conducted a retrospective analysis of patients diagnosed with HCC who had undergone SBRT as part of their disease management between January 2013 and June 2023. Each patient’s case was discussed in a multidisciplinary tumor board comprising experts from various disciplines, including hepatology, medical oncology, hepatobiliary and transplant surgery, interventional and diagnostic radiology, and radiation oncology. Nonsurgical local treatments, including SBRT, RFA, MWA, TACE, and selective internal radiation therapy (SIRT), were offered to patients deemed ineligible for surgical resection, still suitable for local therapy. The choice of SBRT over other treatment options was based on specific factors such as tumor location, including those near the liver dome or blood vessels, lesion size exceeding 3 cm, contraindications for anesthesia, and the need for adjuvant or salvage therapy following TACE.

Only patients who received liver-directed SBRT, as defined by the German Society for Radiation Oncology [5], were included in the analysis. Exclusion criteria were (1) radiation was delivered in symptom palliation, (2) SBRT to lesion outside the liver, (3) none of the survival parameters could be retrieved, and (4) active second malignancy at the time of SBRT application.

A cohort of 54 patients diagnosed with HCC who underwent liver-directed SBRT as part of their disease management was initially identified. However, 2 patients were subsequently excluded from the analysis: one patient was undergoing active treatment for non-small cell lung cancer at the time of SBRT delivery, and no retrievable survival data were accessible for the second patient. Therefore, our final analysis encompassed a total of 52 patients. Among them, comprehensive survival and imaging data from 49 patients, including a total of 62 treated lesions, were available for this study. For the remaining 3 patients, only overall survival data could be analyzed.

### SBRT planning and delivery

First, patients received coaching for inspiration breath-hold (iBH). If patients sustained iBH for 20–30 s, they were considered eligible for simulation and treatment in iBH; if not, they were considered for 4D simulation and treatment.

Briefly, each patient received computer tomography for planning purposes (P-CT) on a 16-slice CT scanner (Brilliance CT Big Bore Oncology, Philips Medical Systems, Inc., Cleveland, OH, USA) in vacuum cushions. Patients received screen goggles connected to an optical surface scanning system (CRAD, Uppsala, Sweden) during the P‑CT and radiation session to facilitate regular breathing in the target zone.

Patients received the three phases of contrast medium-enhanced CT (early arterial, venous, and late venous phases) in the same breathing phase/phases of the P‑CT. If fiducials were necessary, the insertion was applied CT or ultrasound-guided 1 week before P‑CT.

Planning and diagnostic scans were transferred to the planning system (Pinnacle, V.14.0, Philips Healthcare, Amsterdam, The Netherlands). In the case of radiation intrahepatic lesions, gross tumor volume (GTV) was defined as lesions with arterial enhancement and venous washout. In the case of radiation to portal vein tumor thrombus (PVTT), the whole thrombus was considered as GTV, irrespective of enhancing the characteristics. Internal target volume (ITV) was applied for patients who received a 4D CT. In short, contrast-enhanced CTs were deformable, registered with 0%, 50%, and 90% respiratory phase-CTs, and ITV was the sum of arterial enhancement of all three phases. Clinical target volume (CTV) was considered in certain situations as previous TACE of the lesion (including the whole nonenhancing part of the lesion as well) or in the case of SBRT of the recurrent lesion after surgical resection (including the entire resection cavity as well). Planning target volume (PTV) was generated by adding isotopic margins of 5 mm to GTV/ITV/CTV.

The radiation dose was prescribed to enclose PTV in 80% isodose-line to generate the required dose inhomogeneity inside the target volume. Planning aimed to ensure the best coverage of the target volume without compromising the constraints of organs at risk (supplementary table 1), underdosage in the target volume was allowed to ensure the constraints for organs at risk were met. SBRT was delivered as flattening filter-free (FFF) volumetric modulated arc therapy (VMAT). Radiotherapy was delivered 3–4 times weekly as image-guided radiation therapy (IGRT) using cone-beam CT imaging (XVI, Elekta, Stockholm, Sweden) before each fraction for accurate radiation delivery.

### Outcomes evaluation

One month after SBRT, patients were assessed clinically and serologically; this included a complete blood count (CBC), liver function test (LFT), and alpha-fetoprotein (AFP). Imaging assessment using CT or MRI followed 3 months (2 months in case PVTT) after SBRT, and subsequently, every 3 months for 24 months afterward, and finally every 6 months if there were no signs of progression. The radiological response assessment was evaluated based on the modified Response Evaluation Criteria in Solid Tumors (RECIST) criteria for HCC [17]. In summary, complete response (CR) was defined by the complete disappearance of the initial irradiated contrast-enhanced lesion in the arterial phase or tumor disappearance for PVTT (the treated lesion), partial response (PR) was defined as a more than 30% reduction in the size of the treated lesion, stable disease (SD) was defined as reduction size of the treated lesion < 30% or light increase in the size < 20%, and progressive disease (PD) was defined as having an increase of 20% in the size of the treated lesion.

The toxicity was graded using the National Cancer Institute Common Terminology Criteria for Adverse Events (CTAE) version 5.

Radiation-induced liver disease (RILD) is a not-infrequent complication of liver-directed SBRT. For the analysis, we evaluated both forms of RILD. The classical form, cRILD, was defined as anicteric hepatomegaly with ascites and elevated serum alkaline phosphatase more than twice the baseline or upper limit of the normal [18]. The nonclassical form of RILD (ncRILD) was defined as Child–Pugh score (CPS) progression ≥ 2 points or elevation of the alanine transaminases more than four times the upper limit [19].

### Statistical analysis

The primary endpoint of the analysis was freedom from local progression (FFLP), defined at the treated lesion level as the time from radiation initiation until the subsequent local progression or censored. Overall survival (OS) was defined as the interval from the initiation of SBRT to the time of death or censoring. Progression-free survival (PFS) was defined as the interval from initiating the radiation treatment to the point of any site disease progression or censoring. In the case of a liver transplant, FFLP for the respective patient was censored at the time of transplant.

The log-rank test was used for univariate analysis, and the Cox regression was used for independent predictors for PFS and OS. The time-dependent receiver operating characteristic curve (ROC) analysis was applied to calculate the most robust statistical significance cut-point. Mathematical models for FFLP using logistic regression to estimate FFLP probability as a function of prescribed dose (PD) and D98% were created using the radiation dose as EQD2_α/β10_.

1 $$P(\mathrm{Y}=1)=1/\left(1+\mathrm{e}^{-(\upbeta 0+\upbeta 1\mathrm{X})}\right)$$

where

- *P *(Y = 1) is the probability of the event occurring (tumor control),
- e is the base of the natural logarithm,
- β0 is the intercept term in the logistic regression equation, and
- β1 is the coefficient for the independent variable X (radiation dose).

The same was applied for calculating the probability of ncRILD using logistic regression analysis between the mean radiation dose to the liver into EQD2_α/β3_ and EQD2_α/β8_ and CPS progression ≥ 2 points.

The statistical analysis and graphics were executed using the R software version 4.3.1.

## Results

Table 1 provides an overview of patient and disease characteristics. The median follow-up period for the entire cohort was 16.7 months. Sixteen patients had early-stage HCC, and 36 had intermediate and advanced stages (Barcelona clinic liver cancer “BCLC” stage B and C), with 25 patients with BCLC B and C having received prior therapy before SBRT. Sixty-two lesions were evaluable for the analysis with a median volume of 8.15 (0.5–539) cm^3^. The median of the prescribed physical dose was 40 Gy in a median 5 fractions (EQD2_α/β10_ 60 Gy).

**Table 1:** Baseline characteristics

Characteristics	
*Number of evaluable patients*	52
*Number of evaluable lesions*	62
*Volume of lesions (range), *cm^3^	8.15 (0.5–539)
*Median age (range)*	74 (53–93)
*Gender*	
Female	17
Male	35
*BCLC stage*	
A	16
B	27
C	9
*Etiology of liver cirrhosis*	
Alcoholic liver disease	20
Nonalcoholic steatohepatitis	11
Viral hepatitis	6
Other	15
*Child–Pugh score*	
A	33
B	14
C	4
NA	1
*ECOG*	
0–2	41
3–4	11
*PVTT*	
Yes	9
No	43
*Previous treatment*	25
TACE	12
RFA/MWA/IRE	5
Surgery	13
SIRT	1
Systemic therapy	2
*Median physical dose*	40 (24–66) Gy
*Medina dose as EQD2*_*α/β10*_* (range) Gy*	60 (32–87.5) Gy
*Median number of fractions (range)*	5 (3–12)

*BCLC* Barcelona Clinic Liver Cancer staging system, *PVTT* portal vein tumor thrombus, *TACE* transarterial chemoembolization, *RFA* radiofrequency ablation, *MWA* microwave ablation, *SIRT* selective internal radiation therapy, *EQD2* median equivalent dose in 2 Gy per fraction, *Gy* Gray

Notably, 4 patients received SBRT as bridging for a liver transplant and were subsequently transplanted at 1.5, 2.4, 6.2, and 10 months after SBRT. Three patients had a pathological complete response (pCR) at the time of the transplant (2.4, 6.2, and 10 months after SBRT), and 1 patient had a pathological partial response (viable cells in 20% of the lesion 1.5 months after SBRT), the survival of the 4 patients was 9, 10.5, 12.3 and 17.7 months at the time of the analysis (supplementary figure 1).

### Local tumor control

**Fig. 1:**
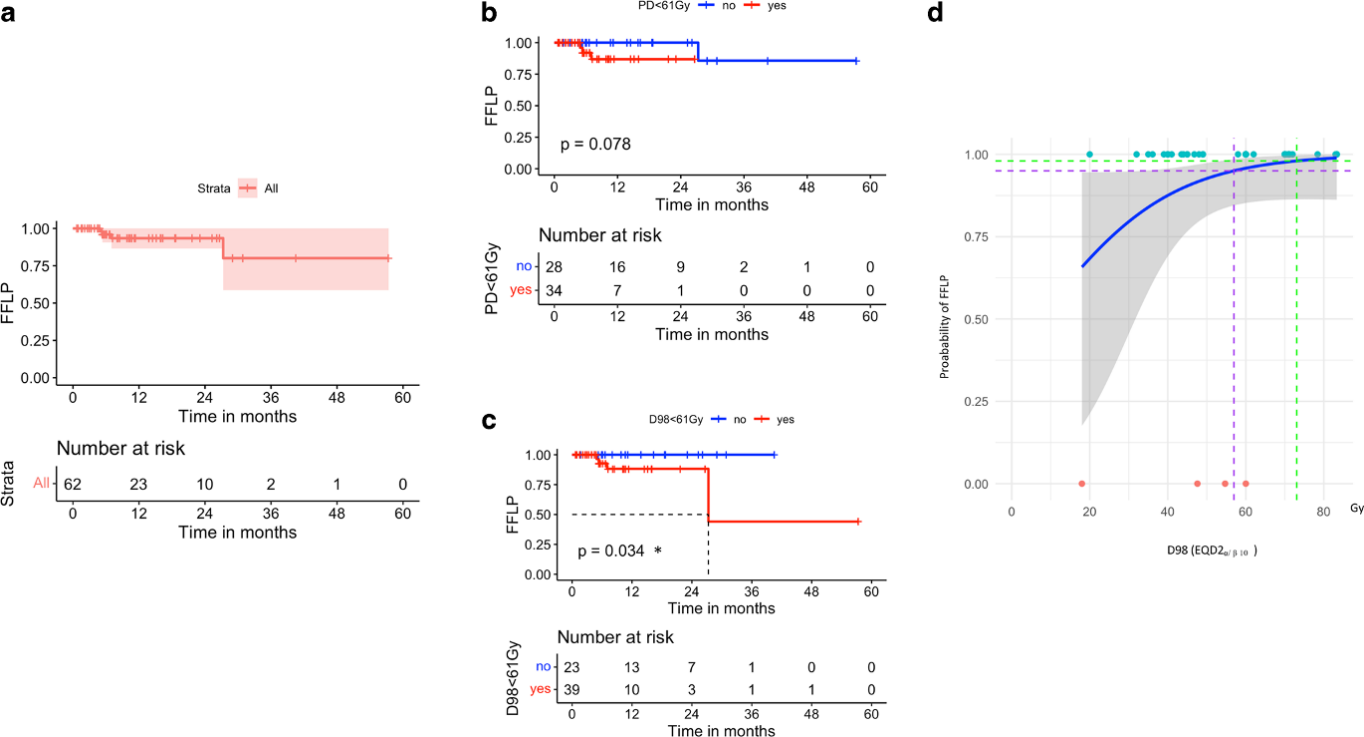
Kaplan–Meier curve showing **a** freedom from local progression (FFLP) for the irradiated lesions, **b** difference in FFLP for lesions with prescribed dose (PD) EQD2_α/β10_ ≥ 61 Gy and < 61 Gy, and **c** the difference in FFLP for lesions with dose near minimum (D98%) EQD2_α/β10_ ≥ 61 Gy and < 61 Gy, *p-value < 0.05: statistically significant, log rank test. **d** FFLP model for irradiated hepatocellular carcinoma (HCC) lesions, the logistic regression curve describes the relationship between local control in 3 years and the dose near minimum (D98) EQD2_α/β10_, *purple* and *green dotted lines* represent the radiation dose that achieves 95%and 98% FFL, respectively

Of the 62 lesions treated with SBRT in 49 patients, the response pattern was as follows: 40 lesions displayed complete responses (CR) at a rate of 64.5%, 10 exhibited partial responses (PR) at 16%, 9 remained in a stable disease (SD) state at 14.5%, and 3 displayed progressive disease (PD) at 5%. In total, four lesions encountered local progression; three experienced direct radiographic local progressions at 4.9, 5.3, and 7 months posttreatment, while one lesion initially showed CR; however, a new lesion or lesion regrowth occurred in the former PTV 27.3 months after treatment. FFLP rates at 12, 24, and 36 months were 93.4%, 93.4%, and 80.1%, respectively (Fig. 1a).

Univariate analysis was conducted to investigate the influence of tumor size, the treatment of intrahepatic lesions versus PVTT on local control, and dosimetric parameters of the SBRT. No statistically meaningful differences existed between small and larger tumors (< 37 vs ≥ 37 cm^3^) or intrahepatic versus PVTT in FFLP. Furthermore, the prescribed radiation dose (PD) in equivalent dose in 2‑Gy fractions (EQD2_α/β10_) to the planning target volume (PTV) below 61 Gy exhibited a trend for association with lower FFLP (*P*-value 0.078; Fig. 1b). Also, examining D98% of PTV showed a D98% < 61 Gy (EQD2_α/β10_), associated with a statistically significant lower FFLP (*P*-value 0.034; Fig. 1c).

In addition, a logistic regression analysis was performed to examine the relationship between dosimetric parameters of the PTV and FFLP.

The initial model, examining PD as a predictor, demonstrated a modest dose–response relationship with the probability of FFLP. The estimated dose coefficient (*β1*) was 0.043 with a corresponding *p*-value of 0.35, suggesting a limited statistical significance. Conversely, the second model, which considered D98% for PTV as a predictor, indicated a more substantial dose–response relationship, *β1* was noted at 0.06 with a *p*-value of 0.085, approaching statistical significance. Using this model, the projected dose to achieve 95 and 98% FFLP were at 56.9 and 73.1 Gy, respectively (Fig. 1d).

### Survival outcomes

**Fig. 2:**
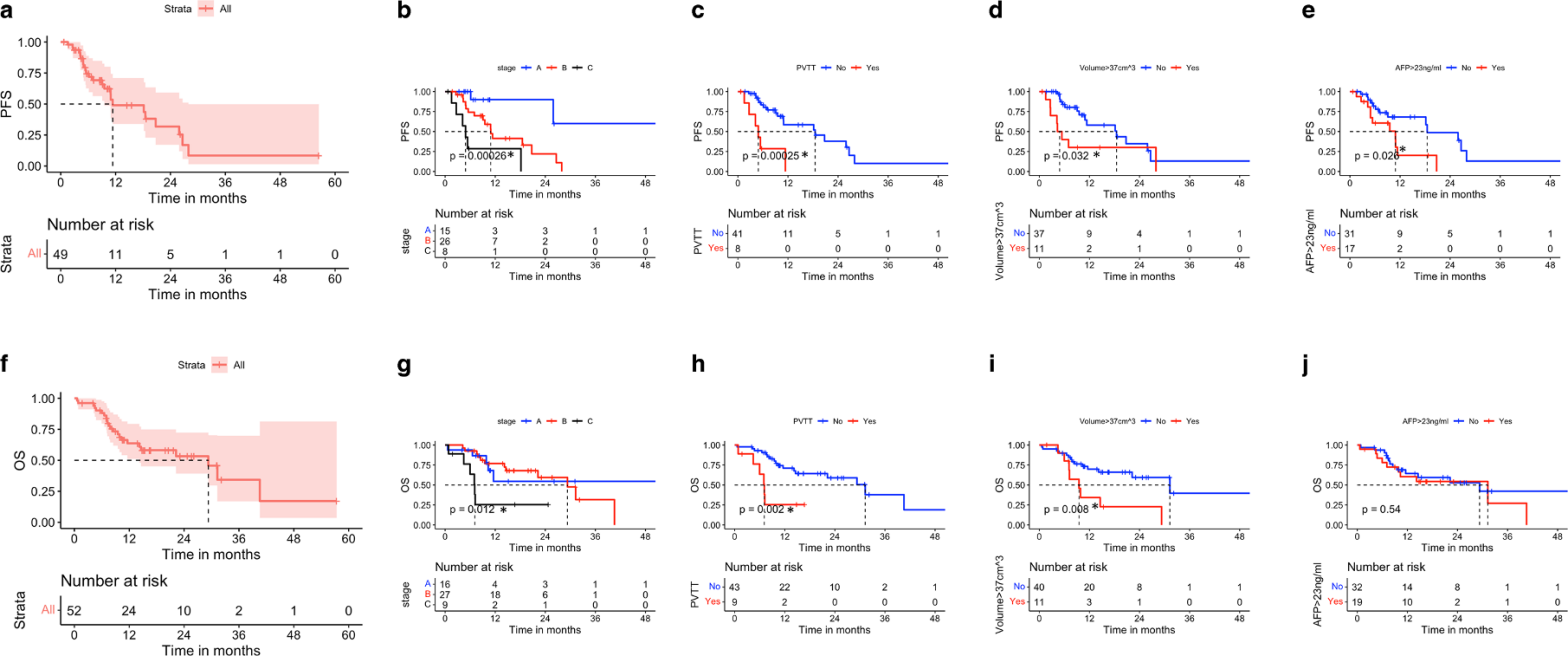
Kaplan–Meier curve shows the progression-free survival (PFS; **a**) and overall survival (OS; **b**) for the entire cohort. Univariate analysis using Kaplan–Meier curves (KM) with log-rank test for PFS and OS based on disease stage (BCLC; **c**, **d**), irradiation of portal vein tumor thrombus (PVTT; **e**, **f**), tumor volume ≥ 37 cm^3^ (**g**, **h**), alpha-fetoprotein level > 23 ng/ml (**i**, **j**). *p-value < 0.05: statistically significant, log-rank test

In all, 17, 3, and 4 patients experienced hepatic, extrahepatic, and hepatic and extrahepatic progressions, respectively, as the first site of progression after SBRT at the time of the analysis with median PFS was 11.4 months (Fig. 2b); a subsequent SBRT after disease progression was applied in 5 patients, TACE in 5 patients, RFA in 2 patients, SIRT in 1 patient, and systemic treatment in 12 patients. The median PFS for stage A was not reached at the time of the analysis, and for stages B and C were 10.9 and 4.9 months, respectively (Fig. 2c).

Twenty-three out of 52 patients had passed away at the time of the analysis, with a median OS of 29.3 months. The OS at 1 and 3 years were 63.7 and 34.3%, respectively (Fig. 2a). The median OS for stage A was not reached, while the median OS for stage B and C was 29.3 and 7.1 months, respectively (Fig. 2c).

A cut-off point for tumor volume (37 cm^3^) and AFP (23 ng/ml) was identified using ROC analysis. In the univariate analysis, SBRT of PVTT or lesions ≥ 37 cm^3^ were associated with statistically significant shorter PFS (*p*-value 0.00025 and 0.032 respectively) and OS (*p*-value 0.002 and 0.008, respectively; Fig. 2c, d, h, i, Table 2). Furthermore, pre-SBRT AFP > 23 ng/ml was associated with PFS (*P*-value 0.026); however, this was not translated into a difference in OS (*p*-value 0.54; Fig. 2e, j, Table 2). Postprogression TACE or systemic therapy did not alter OS compared to those who did not receive those interventions (*p*-value 0.13 and 0.65, respectively, supplementary figure 2; Table 2).

**Table 2:** Univariate (log rank) and Cox regression analyses

	Univariate analysis (log rank): *p*-value		Cox regression: HR (*p*-value)	
	PFS	OS	PFS	OS
ECOG status (0–2 vs 3–4)	0.64	0.0068*	2.2 (0.18)	4.5 (0.002) *
CP score (A vs. B&C)	0.53	0.0078*	–	–
BCLC stage	0.00026*	0.012*	–	–
PVTT	0.00025*	0.002*	4.04 (0.02) *	3.5 (0.046) *
Tumor volume ≥ 37 cm^3^	0.032*	0.008*	2.62 (0.045) *	3.7 (0.012) *
AFP > 23 ng/ml	0.028*	0.54	1.9 (0.2)	1.2 (0.71)
TACE (postprogression)	0.13	0.88	–	–
Systemic therapy (postprogression)	0.00025*	0.65	–	–

*CP* Child–Pugh, *PVTT* portal vein tumor thrombus, *AFP* alpha-fetoprotein

*p-value < 0.05: statistically significant

In the multivariant analysis using Cox regression, a model using ECOG status, PVTT, tumor volume, and AFP for PFS and OS was conducted based on the univariate analysis; the C‑index for the model was 0.72 and 0.75, respectively. PVTT (hazard ratio [HR] 4.04) and tumor volume ≥ 37 cm^3^ (HR 2.62) were associated with shorter PFS. Nonetheless, ECOG status (HR 4.5), tumor volume ≥ 37 cm^3^ (HR 3.7), and PVTT (HR 3.5) were associated with shorter OS (Table 2).

### Change in Child–Pugh score and severe toxicity

CRILD occurred in none of the patients under investigation. However, 4 patients (7.7%) encountered a permanent one-point increase in CPS within the 6 months following SBRT. Conversely, 1 patient (1.9%) experienced a one-point improvement in their CP score after undergoing SBRT. Additionally, 4 patients (7.7%) experienced a two-point progression in CPS within 6 months after SBRT (ncRILD) Tragically, 1 patient (1.9%) with a two-point CPS progression passed away due to hemorrhagic shock resulting from refractory variceal bleeding 3 months after SBRT.

**Fig. 3:**
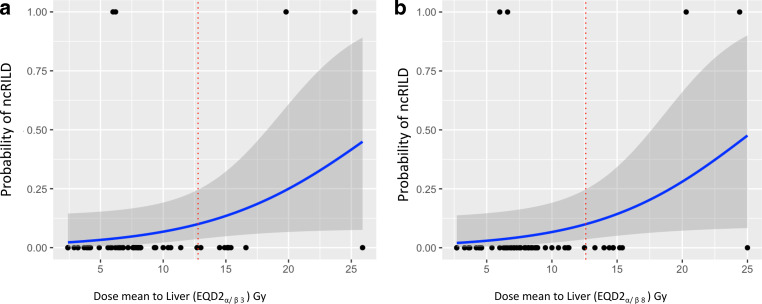
The logistic regression curve describes the relationship between the probability of CPS-P ≥ 2 points “ncRILD” (nonclassical radiation-induced disease) and dose mean to the liver as EQD2_α/β3_ (**a**) and EQD2_α/β8_ (**b**) the *red dotted* line describes the 10% probability function

Further, we performed a logistic regression analysis between the liver mean dose (EQD2_α/β3&8_) and CP score two-point progression (ncRILD) at 6 months [20]. *β1* for the model using EQD2_α/β3_ was 0.15 and approaching the statistical significance (*p*-value 0.06), while *β1* for model with EQD2_α/β8_ was 0.17 and statistically significant (*p*-value 0.048). The analysis revealed a 10% probability of a two-point progression in CPS at 12.8 Gy (EQD2_α/β3_) and 12.6 (EQD2_α/β8_) mean dose to the liver, with an exponential increase in the probability of two-point CPS progression after 15 Gy, reaching an almost 50% probability at 25 Gy (EQD2_α/β3_; Fig. 3). The mean dose delivered to the liver for patients with two-point progression was not statistically significantly higher than for patients with stable or one-point CP progression (suppl. figure 2).

None of the patients experienced biliary stenosis or grade 3 or higher gastrointestinal toxicities.

## Discussion

Previously, the treatment results of HCC with radiation were unsatisfactory due to the limited accuracy in the application of the radiation dose to the target volume, which was compensated with the irradiation of a large volume of healthy liver parenchyma, resulting in high toxicity and a lower response rate [21]. Lately, stereotactic radiotherapy (SRT), initially developed for treating cranial tumors, emerged as one of the curative methods for early HCC, delivering ablative radiation doses to tumors while sparing the adjacent healthy liver tissues and yielding a high local control rate of around 98–90% [22–24]. Comparative studies with thermal ablation showed similar efficacy for small lesions and better results for tumors larger than 3 cm in diameter or the subphrenic area of the liver [15, 25, 26]. Nonetheless, several retrospective and prospective studies showed the superiority of SBRT regarding the local control for HCC compared to TACE [27–30].

The current analysis provides some insights into managing HCC using SBRT. The assessment of local tumor control is paramount when evaluating the efficacy of any local treatment modality for HCC. In this study, the local tumor control achieved with SBRT was convincingly high, with FFLP rates at 1, 2 and 3 years of 93.4%, 93.4%, and 80.1%, respectively, with local progression in only 4 cases, underscoring the potential of SBRT in achieving durable local control. In the univariate analysis, the tumor volume, or intrahepatic tumors versus PVTT did not differ for the local control. Notably, PD as EQD2_α/β10_ ≥ 61 Gy showed a trend for better local control (*p*-value 0.078), and D98 as EQD2_α/β10_ > 61 was associated with a statistically significant improvement of local FFLP (*p*-value 0.034).

Previously, various studies tried to find a dose–response relationship in HCC; some could not establish one [31], while others claimed an advantage for higher doses over lower ones for the local control [32, 33]. In our analysis, the logistic regression model relating PD to FFLP showed a weak dose–response relationship, with *β1* of 0.043 and a nonsignificant *p*-value “0.35”. This suggests a limited direct correlation between PD in the reported dose range (median 40 Gy in 5 fractions: EQD2_α/β10_ : 60 Gy, interquartile range 56–70 Gy) and local tumor control, a finding that aligns with Ohri et al. [31]. The analysis involving D98% presented a more pronounced dose–response curve, with a *β1* of 0.06 and a marginally significant *p*-value “0.085” with TCP values for 95 and 98%, projected at 56.9 and 73.1 Gy, respectively. This enhanced relationship suggests that D98% may serve as a more reliable predictor for local tumor control, potentially providing a more precise dosimetric criterium for the evaluation of the radiation therapy plan in HCC. The discrepancy in the predictive value of PD and D98% could be attributed to the PD parameter not accounting for the spatial distribution of the dose within the PTV, unlike D98%, which is more reflective of the actual dose coverage in the target volume. These considerations are pivotal and may underscore the implication of an individualized dose prescription in radiation therapy for HCC. Based on the current analysis, an EQD2_α/β10_ ≥ 61 Gy (equal to biological effective dose “BED_α/β10_” 73.3 Gy) proved to be sufficient to reach a high probability of tumor control, but a higher EQD2_α/β10_ > 73.1 Gy (BED_α/β10_ 85 Gy) may not translate into a meaningful advantage for FFLP.

These results should be considered in the light of the assessment of the treatment-related toxicity, which is a critical aspect of any therapeutic intervention. In this study, cRILD was not observed, suggesting the safety of SBRT to spare the uninvolved liver parenchyma. However, changes in CPS were noted; namely, 4 patients (7.7%) experienced meaningful CPS progression ≥ 2 points (ncRILD), and 1 of these patients (1.9%) died of refractory variceal bleeding after CPS progression. Dawson et al. suggested that the dose mean to the liver is the most useful parameter in predicting cRILD in liver SBRT, with cRILD not observed when the dose mean to the liver is below 31 Gy [34]. Nonetheless, ncRILD is mostly underestimated in SBRT of HCC, with some studies reporting its incidence around 20% and suggesting a dose mean to liver below 15 Gy to reduce its incidence [35, 36]. We further generated a logistic regression model to predict ncRILD using the dose mean to the liver. The logistic regression model showed a 10% probability of ncRILD at 12.8 and 12.6 Gy, as EQD2_α/β3_ and EQD2_α/β8_, respectively. It is important to note that the dose mean to the liver for patients with CPS B and C was further kept below 8 Gy, with few exceptions at the initial phase of our SBRT experience.

Regarding survival outcomes, the median OS for patients with “BCLC A” was not reached at the time of the analysis, while the “BCLC B” and “C” were 29.3 and 7.1 months, respectively, despite the high local control rate for the treated lesions. We investigated the possible tumor characteristics associated with worse PFS and OS. Predictably, advanced CP score (B and C) or performance status were associated with reduced OS. Also, the presence of PVTT or a larger tumor ≥ 37 cm^3^ strongly predicted shorter PFS and OS. These results may be useful in a number of aspects, such as the proper selection of patients with good performance status who profit from local therapy. Further, the local therapy tool, such as SBRT, alone may not be sufficient for larger tumors or PVTT to achieve intra- and extrahepatic tumor control [37], and a possible combination for these stages (BCLC B or C) with systemic therapy could be advantageous. Indeed, NRG 11112 recently showed that combining SBRT with sorafenib for stage BCLC B and C resulted in a durable response with higher OS and PFS than sorafenib alone [38]. Novel combinations with immunotherapy are currently being investigated with promising results [39].

Also, the utilization of repeated SBRT after hepatic progression was feasible in 5 patients without increased toxicities and aligns with similar previous reports [40].

### Study limitations

There are some limitations of the current study that should be addressed. The main limitation is the retrospective nature of the study, which could make the analysis prone to selection bias and confounding factors that may impact the results. Second, the patient cohort includes heterogenous tumor stages, and a relatively limited number of patients for each stage group implies caution when addressing the study results.

## Conclusion

This study demonstrates the potential of stereotactic body radiotherapy (SBRT) as an effective treatment modality for hepatocellular carcinoma (HCC), achieving promising local control rates with manageable toxicity across different stages of HCC. SBRT with EQD2_α/β10_ 61–73.1 Gy may be sufficient to achieve durable local control and avoid unnecessary radiation dose exposure to the liver. The dose mean to the liver is the main parameter to predict the Child–Pugh score (CPS) progression after SBRT and should be held below 12.8–12.6 Gy (EQD2_α/β3–8_) and may be lower for CPS B and C. Finally, tumors ≥ 37 cm^3^ and portal vein tumor thrombus (PVTT) may profit more from combining the SBRT with systemic therapy to delay the intra- and extrahepatic progression. Current prospective studies are investigating such combinations.

### Supplementary Information

Supplementary table-1: Organ-Specific Constraints Based on Fractionation Schedules

Supplementary figures

## Data Availability

The datasets generated during and/or analyzed during the current study are available from the corresponding author upon reasonable request.
