# Supplementary material for: Managing hepatocellular carcinoma across the stages: efficacy and outcomes of stereotactic body radiotherapy: A retrospective study
Source: Strahlenther Onkol. 2024 Apr 30;200(8):715–24. doi: 10.1007/s00066-024-02235-5 (PMC11272809; doi:10.1007/s00066-024-02235-5)
Supplement: Supplementary file 1 — Supplementary table-1: Organ-Specific Constraints Based on Fractionation Schedules [file 66_2024_2235_MOESM1_ESM.docx]

| Organ at Risk | Parameter | 3 Fractions in Gy | 5 Fractions in Gy | 8 Fractions in Gy | 12 Fractions  In Gy |
| --- | --- | --- | --- | --- | --- |
| Stomach/Duodenum /Jejunum / Ileum | D 0.1 cm^3^ | 30 | 32 | 38.4 | 45 |
|  | D 5cm cm^3^ | 22.5 | 31.5 | 38 | 44.4 |
| Colon | D 1 cm^3^ | 40 | 50 | 60 | 66 |
| Liver  Child-Pugh A/B | D_mean_ | ≤ 15/ 6 | < 20 (preferred ≤ 15) / ≤ 8 | < 22 (preferred ≤ 18) / ≤ 10-8 | < 24 /NA |
| Common bile duct | D 0.1 cm^3^ | 35 | 40 | 42.2 | 49.8 |

Supplementally, table 1 dose constraints to organs at risk

Supplementary figures

Figur1:

Kaplan Meier curve shows the progression-free survival (PFS) “a” and overall survival (OS) “b” for the patients who underwent transplant after SBRT compared to the entire cohort.

Figure 2:

Univariate analysis using Kaplan Meier curves (KM) with log rank test for progression-free survival (PFS) and overall survival (OS) based on Child Pugh score “ a & b”, performance status “c & d” , TACE after progression “e & f”, Systemic therapy after progression “ g & h”. * p-value < 0.05: statistically significant, log rank test.

Figure 3:

Boxplot shows the mean liver dose for patients with Child-Pugh score (CPS) progression ≥ 2 points “grey” versus those with CPS- stable or 1-point progression “a”.
