# Supplementary figures and images for: Managing hepatocellular carcinoma across the stages: efficacy and outcomes of stereotactic body radiotherapy: A retrospective study
Source: Strahlenther Onkol. 2024 Apr 30;200(8):715–24. doi: 10.1007/s00066-024-02235-5 (PMC11272809; doi:10.1007/s00066-024-02235-5)

# Supplementary Figures

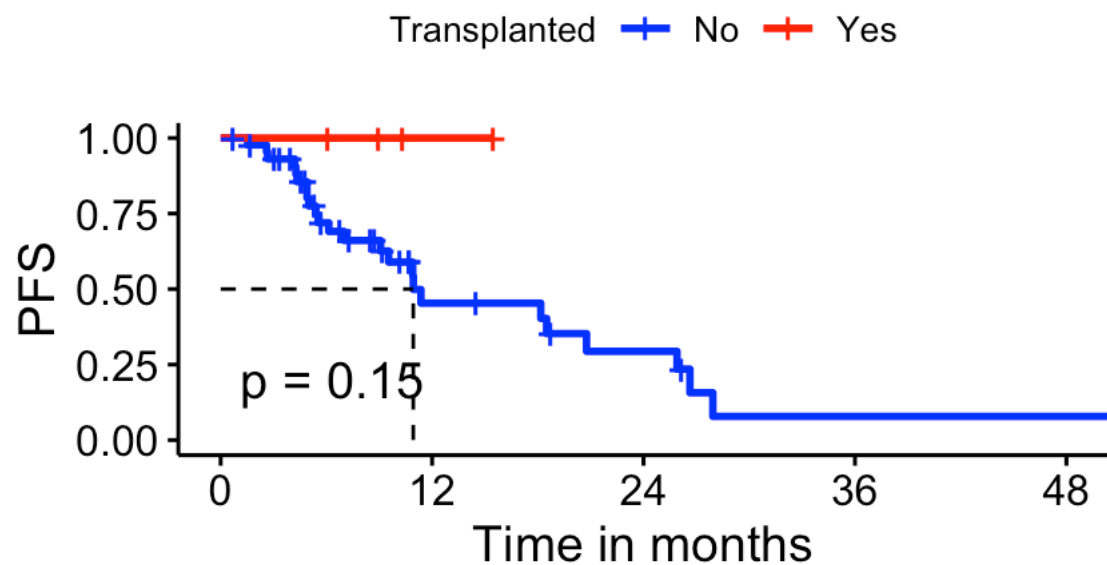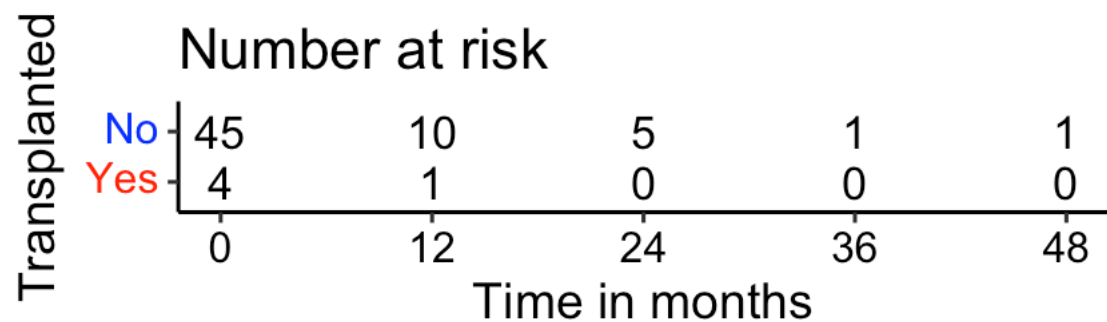

a

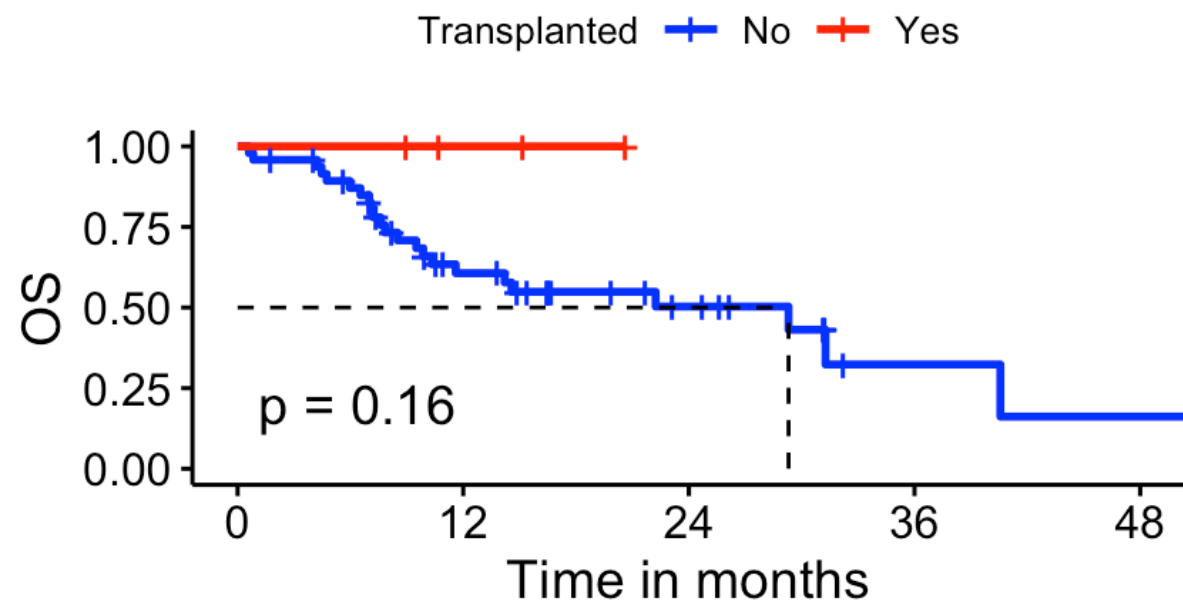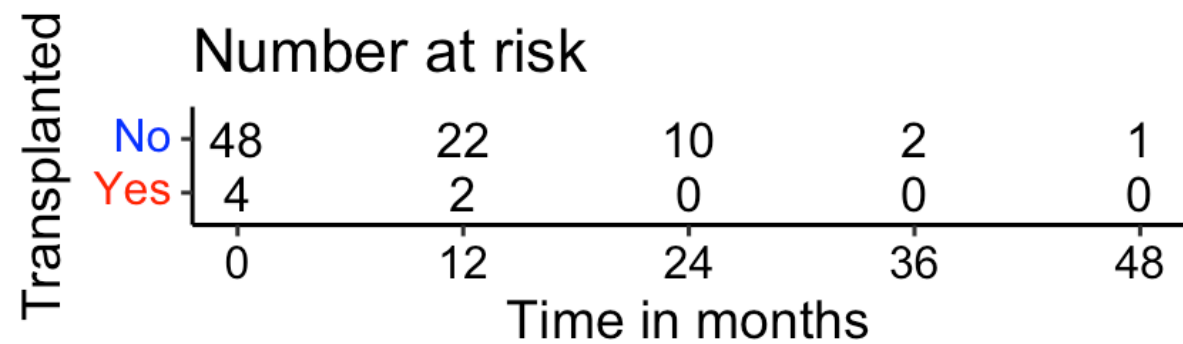

b

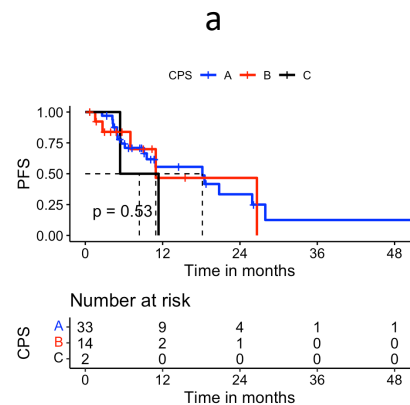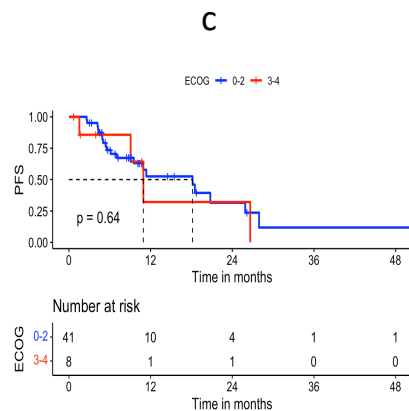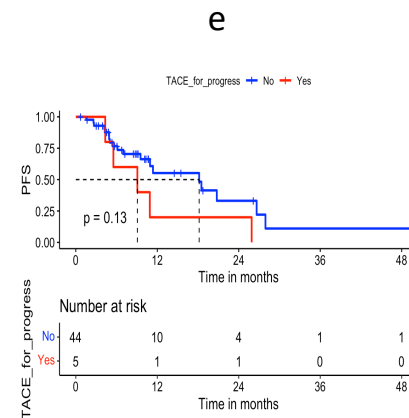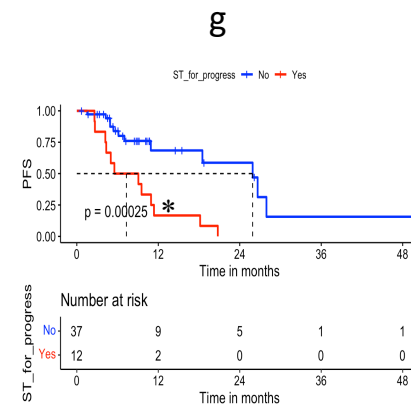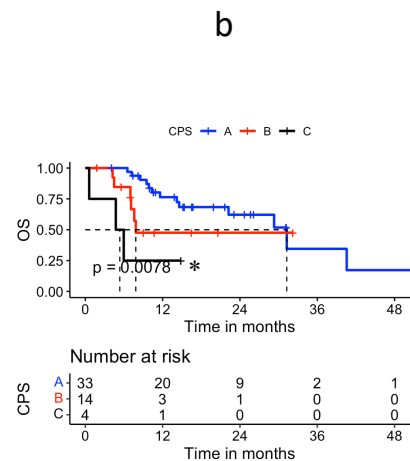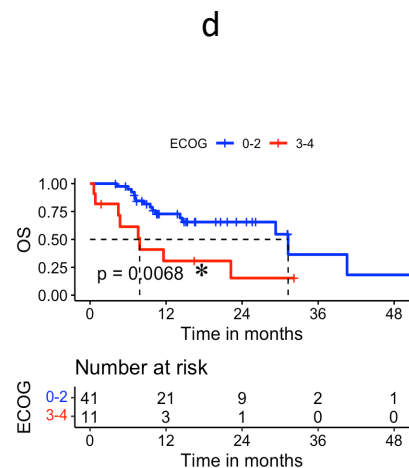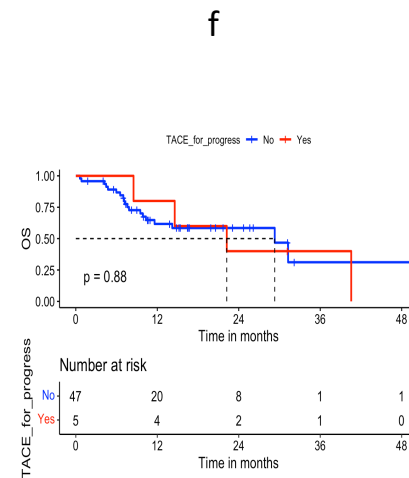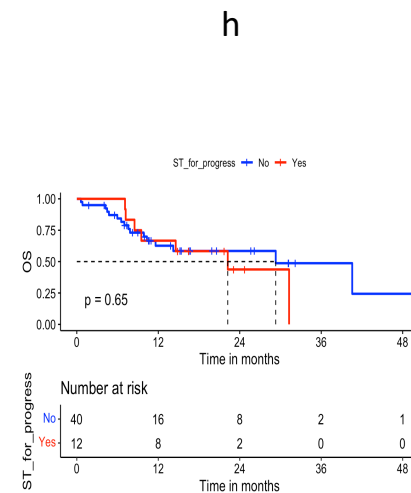

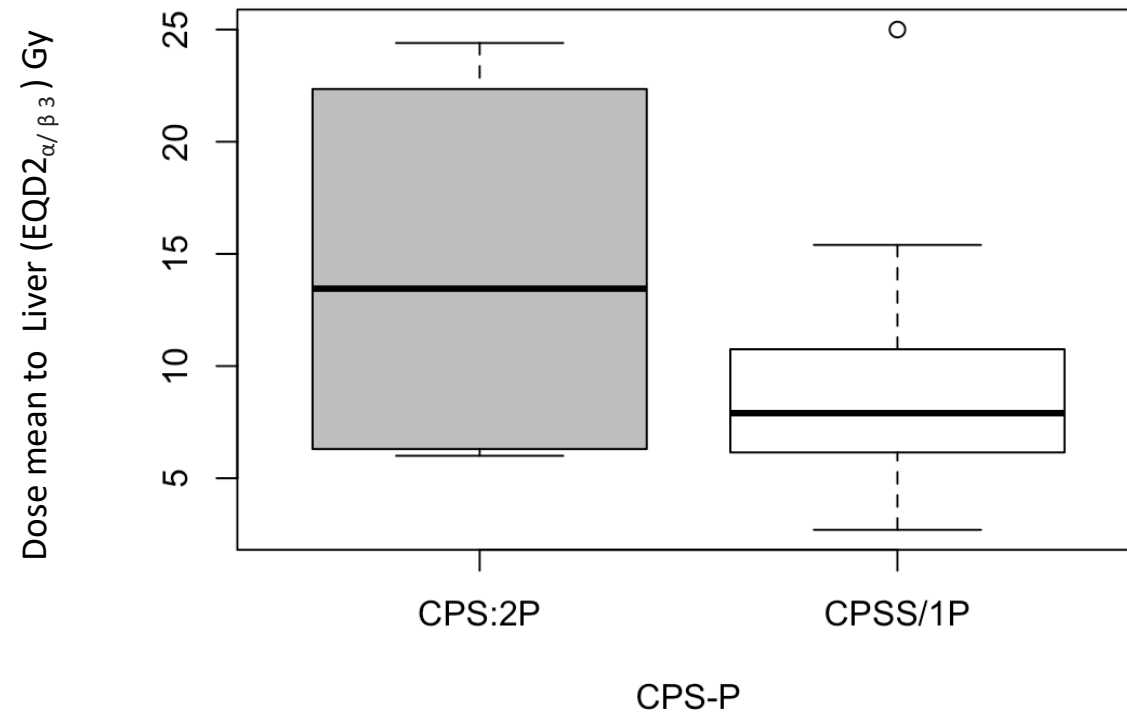

Supplement: Supplementary file 2 — Supplementary figures [file 66_2024_2235_MOESM2_ESM.pdf]
